# Phenotypes and malignancy risk of different *FUS* mutations in genetic amyotrophic lateral sclerosis

**DOI:** 10.1002/acn3.50930

**Published:** 2019-11-04

**Authors:** Marcel Naumann, Kevin Peikert, Rene Günther, Anneke J. van der Kooi, Eleonora Aronica, Annemarie Hübers, Veronique Danel, Philippe Corcia, Francisco Pan‐Montojo, Sebahattin Cirak, Göknur Haliloglu, Albert C. Ludolph, Anand Goswami, Peter M. Andersen, Johannes Prudlo, Florian Wegner, Philip Van Damme, Jochen H. Weishaupt, Andreas Hermann

**Affiliations:** ^1^ Department of Neurology Technische Universität Dresden Dresden Germany; ^2^ German Center for Neurodegenerative Diseases (DZNE) Dresden Germany; ^3^ Translational Neurodegeneration Section “Albrecht‐Kossel” Department of Neurology University Medical Center Rostock University of Rostock Rostock 18147 Germany; ^4^ Department of Neurology Amsterdam UMC Academic Medical Centre Amsterdam Neuroscience University of Amsterdam Amsterdam the Netherlands; ^5^ Amsterdam UMC Department of (Neuro)Pathology Amsterdam Neuroscience University of Amsterdam Amsterdam The Netherlands; ^6^ Department of Neurology German Center for Neurodegenerative Diseases University of Ulm Ulm Germany; ^7^ Centre expert pour la SLA et les maladies du motoneurone hôpital SALENGRO CHU Lille France; ^8^ Department of Neurology Klinikum der Universität München Munich Cluster for Systems Neurology SyNergy Munich 81377 Germany; ^9^ Division of Pediatric Neurology Department of Pediatrics University Hospital Cologne Cologne Germany; ^10^ Center for Molecular Medicine University of Cologne Cologne Germany; ^11^ Department of Pediatric Neurology Hacettepe University Children’s Hospital Ankara 06100 Turkey; ^12^ Institute of Neuropathology RWTH Aachen University Hospital Aachen 3052074 Germany; ^13^ Institute of Pharmacology and Clinical Neuroscience Umeå University Umeå SE‐90185 Sweden; ^14^ German Center for Neurodegenerative Diseases (DZNE) Rostock/Greifswald Rostock 18147 Germany; ^15^ Department of Neurology University of Rostock Rostock Germany; ^16^ Department of Neurology Hannover Medical School Hannover Germany; ^17^ Department of Neurology University Hospitals Leuven Leuven Belgium; ^18^ Department for Neuroscience VIB‐KU Leuven Center for Brain & Disease Research Leuven Belgium; ^19^ Center for Transdisciplinary Neurosciences Rostock (CTNR) University Medical Center Rostock University of Rostock Rostock 18147 Germany

## Abstract

**Objective:**

Mutations in Fused in Sarcoma (*FUS or TLS*) are the fourth most prevalent in Western European familial amyotrophic lateral sclerosis (ALS) populations and have been associated with causing both early and very late disease onset. FUS aggregation, DNA repair deficiency, and genomic instability are contributors to the pathophysiology of FUS‐ALS, but their clinical significance per se and their influence on the clinical variability have yet to be sufficiently investigated. The aim of this study was to analyze genotype–phenotype correlations and malignancy rates in a newly compiled FUS‐ALS cohort.

**Methods:**

We cross‐sectionally reviewed FUS‐ALS patient histories in a multicenter cohort with 36 novel cases and did a meta‐analysis of published FUS‐ALS cases reporting the largest genotype–phenotype correlation of FUS‐ALS.

**Results:**

The age of onset (median 39 years, range 11–80) was positively correlated with the disease duration. C‐terminal domain mutations were found in 90%. Among all, P525L and truncating/ frameshift mutations most frequently caused juvenile onset, rapid disease progression, and atypical ALS often associated with negative family history while the R521 mutation site was associated with late disease onset and pure spinal phenotype. Malignancies were found in one of 40 patients.

**Interpretation:**

We report the largest genotype–phenotype correlation of FUS‐ALS, which enables a careful prediction of the clinical course in newly diagnosed patients. In this cohort, FUS‐ALS patients did not have an increased risk for malignant diseases.

## Introduction

Amyotrophic lateral sclerosis (ALS) is recognized as one of the most severe neurodegenerative diseases. Progressive muscular paresis due to motor neuron (MN) demise leads to a rapid loss of autonomous mobility and usually culminates in death of patients after 3–5 years.^1^ Approximately, 10% of all ALS patients self‐report a familial predisposition. Mutations in more than 38 genes were identified to be implicated in the pathological MN degeneration.^2^ In 1% of sporadic and up to 5% of familial ALS cases, mutations in *Fused in Sarcoma (FUS)* were found to be causative,^3^ primarily located in the PY‐nuclear localization sequence (NLS) of the protein and are often associated with a more severe course compared to patients with C9ORF72, TBK1, TARDBP, or SOD1 mutations. Furthermore, the severity of these genetic errors appears to positively correlate with disease onset^4^ and recently, *FUS* mutations were shown to have the highest proportion of all ALS‐related gene mutations in juvenile ALS patients in Germany.^5^ Indeed, the youngest FUS‐ALS patient reported a disease onset at 11 years of age^6^ presenting with the P525L mutation. In smaller case series, bulbar disease onset was reported more often,^7^ which is known to be a negative predictor for survival in sporadic ALS.^8^ Others, however, reported on FUS‐ALS patients with particularly late onset.^7^, ^9^, ^10^ Altogether, the rarity and variations of individual disease courses make appropriate predictions about individual patient survival impossible.

FUS exerts its function as a DNA‐/RNA‐binding protein primarily in the nucleus and is centrally implicated in splicing regulation, stress granule formation, and DNA repair.^11^, ^12^ We and others recently reported on drastically increased DNA damage in various cell types with *FUS*‐NLS mutations, which was shown to be associated with neuronal cell death.^13^, ^14^, ^15^ It is, however, not known if other cells in FUS‐ALS patients are also affected by accumulated DNA damage leading to genomic instability, which is the basis for the multistep process of cancer development. The fact that *FUS* knock‐out mouse embryonic fibroblasts display abundant chromosomal instability and enhanced radiation sensitivity would underline this reasoning.^16^, ^17^

Neurodegenerative diseases in general were reported to correlate with an altered risk for malignant diseases. Evidence exists for a lower tumor risk in Alzheimer's, Huntington's, and Parkinson's diseases,^18^, ^19^ whereas no difference could be found in overall ALS patients compared to the general population.^20^ However, due to the low prevalence of *FUS* mutations, a possible cancer hazard could be masked. Therefore, we identified via a multicenter approach 36 novel patients with FUS mutations and reviewed available medical records for the presence of malignancies. Furthermore, we analyzed individual disease parameters in context of previously reported FUS‐ALS cases to deepen our knowledge of genotype–phenotype correlations in this rare disease.

## Materials & Methods

The study was performed according to the Declaration of Helsinki and approved by the local institutional review boards (EK 393122012, EK 49022016 at the Technische Universität Dresden). We performed a multicenter cross‐sectional study to identify genetically proven FUS‐ALS patients according to El‐Escorial criteria^21^ and surveyed all available medical records including postmortem (*n* = 8) analysis for the occurrence of neoplasms (Table 1). If applicable, informed consent was obtained from the individuals. Documentation of benign hyperplasia or dysplasia was included into the table but otherwise disregarded because of mostly missing further pathological information. For statistical testing, we compared with the German cancer statistics from 2004^22^ serving as a control group whilst taking the negligible amount of ALS patients therein into account.

**Table 1:** Demographic data of newly reported FUS‐ALS cases (=cohort 1).

Sex	AoO (y)	Age at death (y)	Site of onset	Family history	Amino acid change	Tumor	Tumor in Autopsy	Onset to death (m)	OTSE (m)
female	22	alive	arms	positive	P525L	none reported	not applicable	not applicable	7
female	58	58	arms	positive	R521L	none reported	NA	7	7
female	24	26	bulbar	negative	Y526C	none reported	NA	22	9
female	NA	16	NA	negative	R495*	none reported	negative	10	10
female	33	35	dropped head	NA	Y526C	cystic tumor intraspinal	positive	16	16
female	39	40	right leg	positive	R521C	several benign tumors	positive	19	19
female	38	40	legs	NA	R521C	Focal nodular hyperplasia liver	positive	25	25
female	38	41	legs (right)	positive	R521H^1^	none reported	NA	31	31
female	46	49	arms	positive	R521H	none reported	NA	37	37
female^4^	60	63	arms (left)	positive	R521H^2^	none reported	NA	37	37
female	44	47	arms (left)	positive	R521H^2^	none reported	NA	38	38
female	61	66	arms	positive	R521C	none reported	NA	60	60
female^4^	33	39	legs (left)	positive	R521H^2^	none reported	NA	71	71
female	NA	NA	NA	NA	R521H	none reported	NA	NA	NA
female	NA	NA	NA	NA	R521H	none reported	NA	NA	NA
female	NA	NA	NA	NA	R521H	none reported	NA	NA	NA
female	NA	70	NA	NA	R521C	none reported	NA	NA	NA
female	NA	70	NA	NA	R521C	several benign tumors	positive	NA	NA
female	17	18	bulbar	negative	P525L	none reported	NA	24	24
male	17	18	legs	negative	P525L	none reported	NA	15	15
male	31	32	bulbar	positive	R495Qfs*527	Acute lymphoblastic leukemia (ALL)	NA	18	18
male	23	25	Legs right	positive	G478Lfs*23	none reported	not applicable	19	19
male	39	40	right arm	positive	R521C	none reported	negative	20	20
male	39	41	bulbar	NA	R521C	none reported	negative	20	20
male	54	56	left (arm)	positive	R521C	none reported	negative	27	27
male	71	74	arms (left)	positive	R521H^1^	none reported	NA	29	29
male	62	65	legs (left)	positive	R521C	none reported	NA	48	48
male	43	alive	arms (right)	positive	R521H	none reported	not applicable	not applicable	61
male	63	alive	NA	positive	M254I	none reported	NA	NA	72
male^4^	65	73	arms	positive	R521H^2^	none reported	NA	86	86
male	35	49	right hand	negative	Q23L	none reported	negative	175	175
male	NA	NA	NA	NA	R521H	none reported	NA	NA	NA
male	NA	40	NA	NA	R521C	none reported	NA	NA	NA
male	27	28	arms/ shoulders	positive	R521C^3^	none reported	NA	13	13
male	40	41	arms (left)	positive	R521C^3^	none reported	NA	13	13
male	59	alive	legs (left)	positive	K510R	none reported	not applicable	not applicable	not applicable
male	40	alive	legs (right)	NA	R521H	none reported	not applicable	not applicable	not applicable
male	41	alive	legs	positive	G509D	none reported	not applicable	not applicable	not applicable
male	13	alive	arms	NA	Y526C	none reported	not applicable	not applicable	30

NA, not available, ^1,2,3^indicate familial relation, ^4^indicates single patients that have been already published,^30^ but were included to demonstrate familial relation to others in the table.

Furthermore, all available demographic and disease‐related data were obtained. To measure the individual disease course/survival more precisely, the term “onset to severe event” (OTSE, months, m) was designed estimating the time from onset to the constant need for assisted ventilation, tube feeding, or death, which was also the censoring date for the Kaplan‐Meier curve in Figure 3.

Additionally, 150 published ALS cases with *FUS *mutation (cohort 2) were collected by searching the pubmed database for the terms “FUS,” “ALS,” and “FUS MUTATION.” Only affected patients with both existing clinical data and known mutations status were included. Combined mean or median data were likewise excluded. Using this approach, a total of 186 patients from 44 studies were included in our analysis (Table S1).

### Statistics

Testing for statistical significance and general descriptive analysis was done using the IBM SPSS Statistics version 25 software. Individual tests are described beneath the respective figures, all were carried out as two‐sided test and a *P* ≤ 0.05 was considered to indicate significant test results. The median was used as main data aggregation estimator with the median 95% confidence interval to indicate dispersion. Testing for normality distribution was carried out using the Shapiro‐Wilk test. Normal distribution was found if not stated otherwise in the results section allowing the usage of student *t*‐test. However, for all data depicted by boxplot diagrams, the null hypothesis was rejected, hence either the Kruskal‐Wallis H or Mann‐Whitney U test were used (Fig. 3A, 4B and C). Bonferroni correction was applied in Figure 3A. Following Kaplan‐Meier plotting, the Log‐rank test was used to estimate survival differences between FUS‐ALS patient subgroups. Pearson's Chi‐square test was carried out to evaluate the sex difference frequency. Fisher's exact test was applied to compare the tumor prevalence data from different populations. Spearman rank correlation coefficients were used to examine correlations between age of onset (AoO) and OTSE with a correlation coefficient of rho < 0.3 considered as a weak, rho = 0.3–0.59 a moderate, and rho ≥ 0.6 a strong correlation.

### Data Availability Statement

The authors state that all data are available upon individual request.

## Results

### Demographic and disease‐related data of identified patients with *FUS *mutation

We included two cohorts of FUS‐ALS patients in this study. First, we retrospectively analyzed case files in a cross‐sectional multicentric study and identified 36 unpublished FUS‐ALS cases (Table 1). Additionally, we collected information on recently published FUS‐ALS patients for whom both relevant clinical information and genetic testing were available (Table S1). Demographic data are shown in Tables S2–S4.

In cohort 1, 34 patients (94%) had a C‐terminal mutation within the nuclear localization signal (NLS), whereas the two remaining patients showed mutations at position 23 in the Prion‐like domain (PLD) or at 254 in the Glycine‐rich repeats domain underlining the previously reported abundancy of C‐terminal mutations in FUS patients. Furthermore, our cohort included three cases with the P525L change, four patients were found to have a truncating mutation following inclusion of an early stop codon or frameshift alterations. Male sex was slightly more frequent with a ratio of 1.18:1 (*P* = 0.74, Chi‐square test). The median AoO was 39 (CI 31–44) years and the median survival time from symptom onset until either permanent necessity for live‐prolonging measures or death (OTSE) was 25 months (CI 18–31) months. Sex did neither influence AoO (*P* = 0.8, students *t*‐test) nor OTSE (*P* = 0.26, students *t*‐test). Bulbar disease onset was observed in 13.8% of patients.

Cohort 2 included recently published FUS‐ALS cases for whom clinical information was available and genetic testing was done on the reported individual. By doing so, we collected 150 additional cases (Table S1). C‐terminal domain mutations were found in 89% with the R521C amino acid change being the most prevalent mutation (17%). In general, the locus 521 had the highest abundancy of different amino acid changes and was mutated most frequently (36%). Importantly, with regard to median AoO/OTSE, we observed almost identical values in cohort 2 (Table S3) compared to cohort 1 (Table S2), highlighting the validity of the data (*P* = 0.84 and *P* = 0.61, students *t*‐test, respectively). Due to lack of data availability, the sex influence could not be completely evaluated.

### Genotype–phenotype correlations

**Figure 1:**
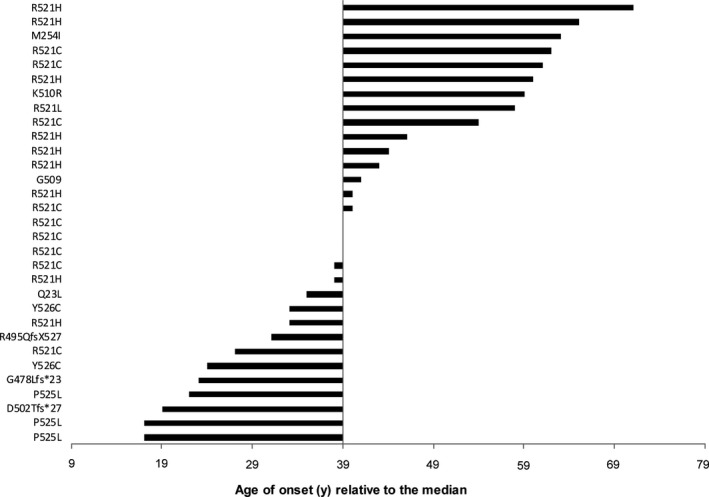
Age at onset of newly acquired patients (=cohort 1) depicted as bar diagram for every case starting from the median value, *n* = 30. Similarly, data of all patients (cohort 1 and 2) are shown in Figure S1.

Figure 1 demonstrates the variability of the age at onset in cohort 1 (see Fig. S1/ DataS1 for all patients) implying that certain mutations do result in very different phenotypes.

As shown above, cohort 1 and 2 did not differ concerning AoO and OTSE (Tables S2–S4). Therefore, we combined the data for further analysis of genotype–phenotype correlations resulting in the so far largest reported cohort of 186 FUS‐ALS patients (Table S1).

**Figure 2:**
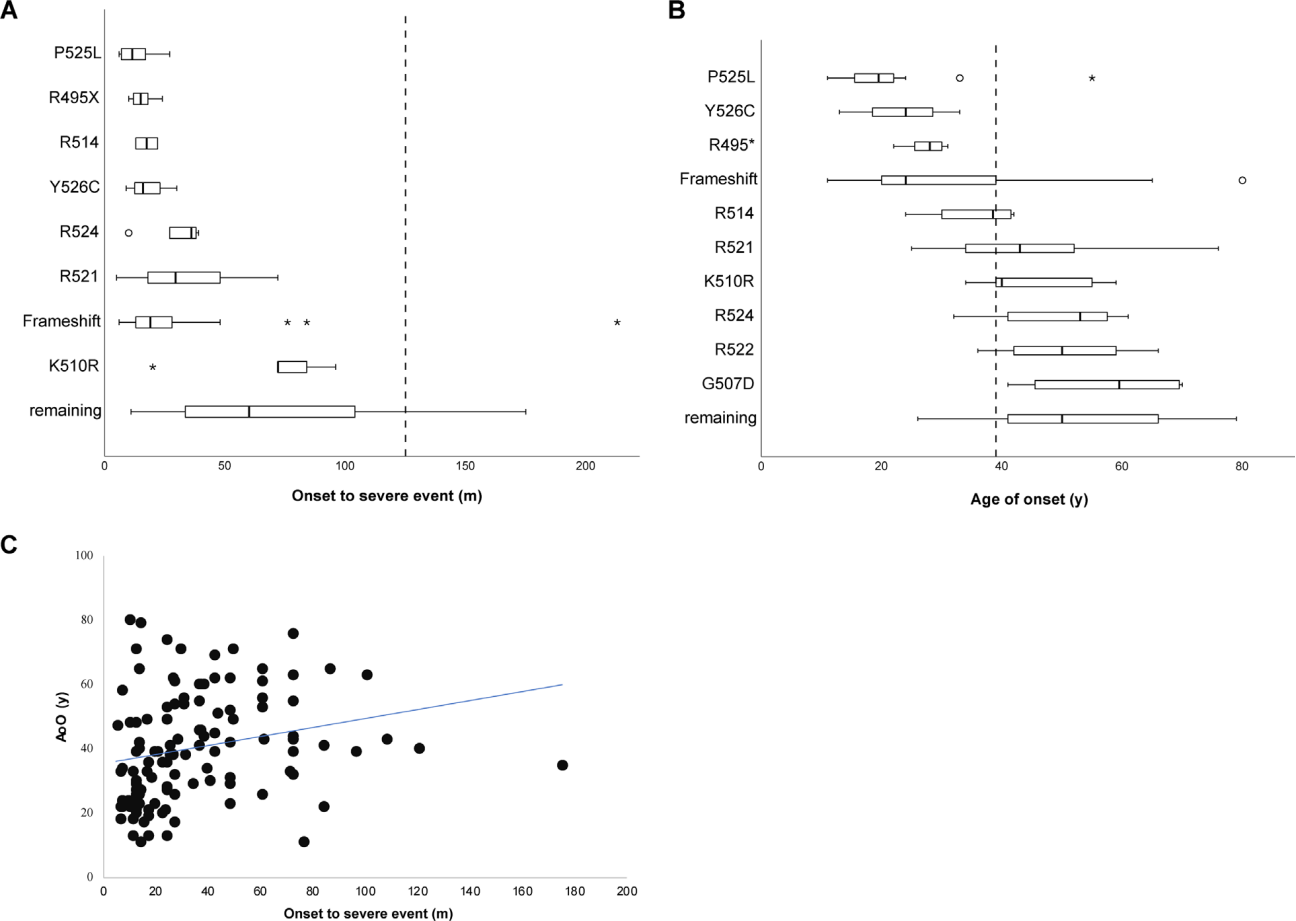
(A), (B) Boxplot diagrams of disease duration (onset to severe event, OTSE) and age of onset (AoO) depending on individual mutation sites in the combined group. The median value of the total group is drawn as broken line. Mutation sites were selected if information was available on ≥ 2 cases. (C) Scatter blots of AoO and OTSE indicating moderate positive correlation.

Table S4 summarizes descriptive data of the combined cohorts. Interestingly, we found a moderate but significant positive correlation between AoO and OTSE (Fig. 2C, Spearman ρ = 0.37, *P* < 0.001), which is contrary to sporadic ALS, in which late onset is associated with faster disease progression.^23^, ^24^

Figure 2 implicates that certain mutations may result in distinct clinical disease parameters. In detail, P525L, Y526C, and R495X led to the most striking difference compared to the other groups listed in Table 2. One patient of the new cohort 1 had a particularly early onset at the age of 13 and subsequent genome sequencing revealed the Y526C missense mutation in his case. On examination, he showed weakness in all extremities without obvious clinical signs of upper MN impairment. Furthermore, he had a mild intellectual disability and a cerebellar nystagmus; however, there was no evidence for bulbar disease. His condition deteriorated rapidly resulting in the need for constant ventilation support and tube feeding 30 months after onset paralleled by locked‐in syndrome.

**Table 2:** Descriptive data of frequent FUS mutation carriers in the combined group.

	R521	R495*	Frameshift	R514	P525L	R524	G507D	K510R	R522	Y526C	Remaining	Total
AoO (y)	43 (39–48, *N* = 69)	28 (24–31, *N* = 8)	26 (22–31, *N* = 28)	39 (36–42, *N* = 4)	19.5 (13–22, *N* = 20)	55 (34–61, *N* = 7)	59.5 (34.7–80, *N* = 4)	40 (39–55, *N* = 8)	50 (30.8–70.2, *N* = 4)	28.5 (24–33, *N* = 3)	47 (34–72.5, *N* = 21)	39 (33.5–41, *N* = 176)
OTSE (m)	31 (25–40, *N* = 54)	18 (12–48, *N* = 6)	19 (12.5–26, *N* = 21)	17.5 (13–22, *N* = 2)	13 (10–20) *N* = 14)	36 (10–39, *N* = 5)	42 (*N* = 1)	78 (72–102, *N* = 6)	24 (*N* = 1)	19 (16–22. *N* = 3)	54.5 (24–108, *N* = 11)	24 (20–28.5, *N* = 124)

Median values (95% Confidence interval of the median) for the age of onset (AoO) and survival time until severe event (OTSE) are shown for patient groups with given mutation patterns.

**Figure 3:**
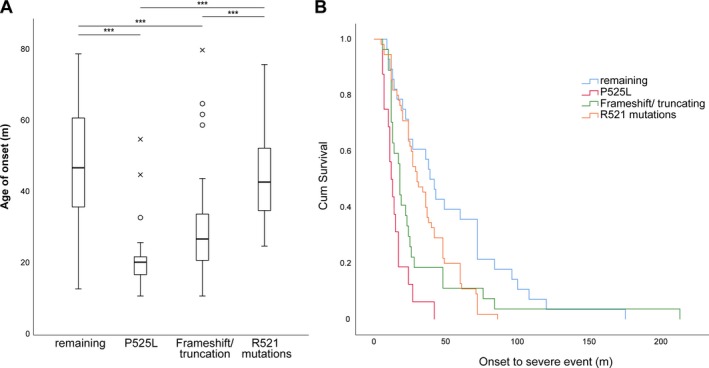
(A) Box plots showing the median age of onset for selected patient groups with the highest frequencies in the cohort. Statistical testing was performed using a Kruskal‐Wallis test followed by Bonferroni correction, ***P < 0.001. (B) Kaplan‐Meier survival curve measuring the OTSE. The groups were found to have significantly different cum. survival rates as demonstrated by the Log‐rank test, *P* < 0.001.

Next, we grouped the most frequently reported amino acid changes at the loci P525L, R521, truncating/frameshift mutations, and others for deeper investigation. Figure 3A demonstrates that carriers of a P525L (21 years, CI 15–22) or truncating/frameshift (27 years, CI 23–31) mutation had a significantly lower median age of onset compared to the remaining carriers (47 years, CI 39–55) and to the R521 carriers (43 years, CI 39–49, Kruskal‐Wallis H test, post hoc Bonferroni correction, *P* < 0.001). Interestingly, the cumulative survival of the individual mutation carriers was significantly different (Fig. 3B, Log‐rank Test, df = 3, *P* < 0.001) as indicated by the Kaplan‐Meier curves demonstrating the shortest survival for the P525L patients.

**Figure 4:**
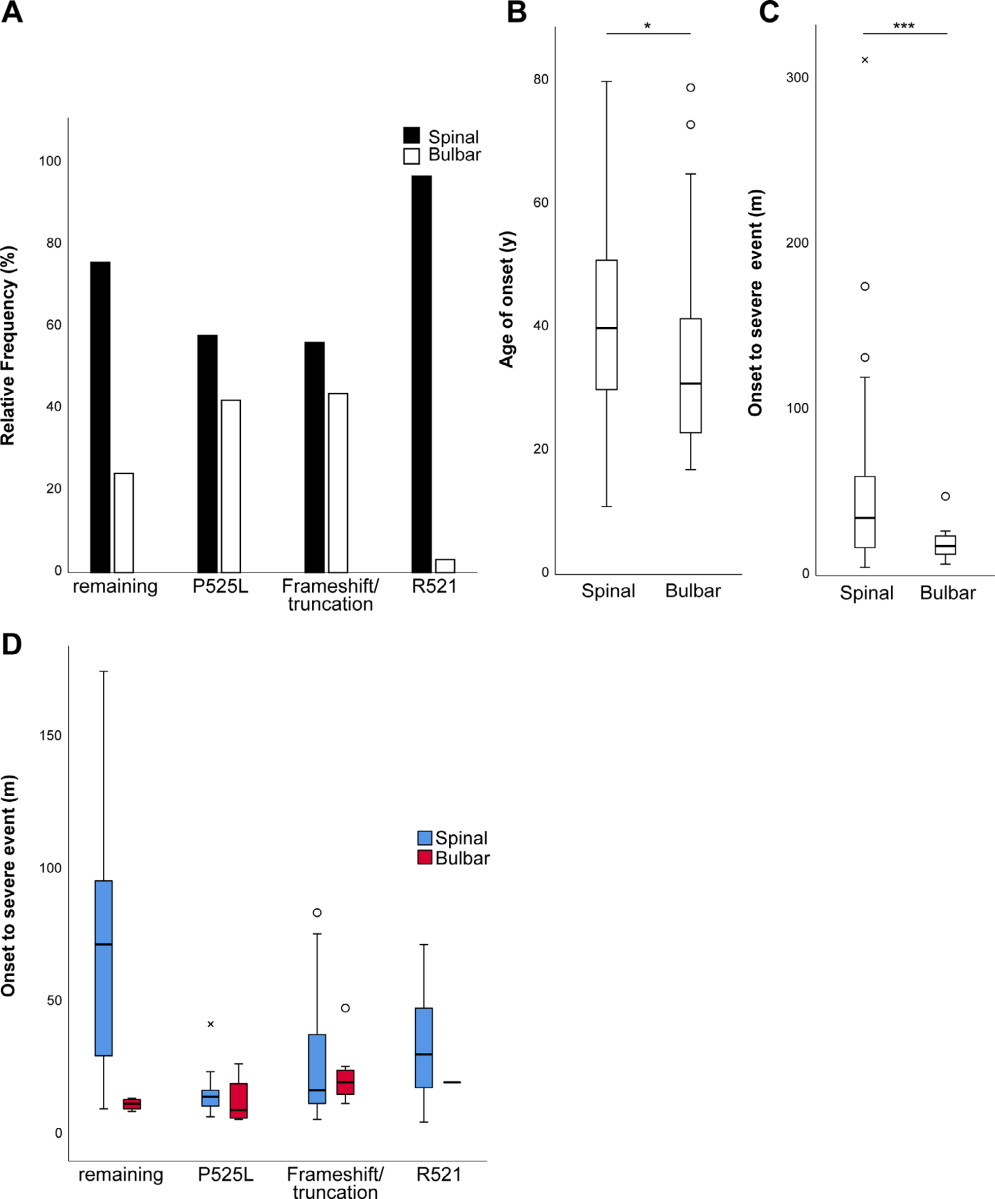
(A) Percentage of spinal vs. bulbar onset for the four most prevalent groups. Note the high rate of patients with a P525L or frameshift/truncating mutation presenting with bulbar disease, whereas R521 carriers mostly show spinal disease course. (B) Median age of onset and (C) OTSE grouped by spinal or bulbar disease onset. FUS‐ALS patients with bulbar onset had a significantly lower age of onset and survival as revealed by Mann‐Whitney U test. (D) Median survival time for selected mutation carriers grouped by side of onset. *P < 0.05, ***P < 0.001.

Further analysis of the data revealed that the initial site of disease onset is different for individual mutations (Fig. 4A). Whereas P525L (42%) and truncation/frameshift (44%) carriers (44%) more frequently presented with initial bulbar disease, only 3% of R521 mutation carriers had such symptoms at onset. As expected, we observed a significantly shorter median OTSE in bulbar‐onset FUS‐ALS patients compared to patients with spinal onset (18 m, CI 13.8–22.3 vs. 30 m, CI 31.8–44.8, *P* = 0.002, Mann‐Whitney U test). Surprisingly, however, the patients with bulbar course were additionally present with a significantly lower median age of onset (31 years, CI 24–36 vs. 40 years, CI 36–43, *P* = 0.025, Mann‐Whitney U test, Fig. 4B and C).

Considering that the site of initial symptoms had a significant effect on both AoO and OTSE, one could deduce that the higher proportion of patients with bulbar disease might result in the decreased OTSE and earlier age at onset for the selected groups in Figure 3. However, when analyzing the OTSE for bulbar or spinal patients (Fig. 4D), no clear differences became obvious suggesting that additional modifiers might influence the disease phenotype, especially in the P525L group.

### Analysis of malignancy burden in FUS‐ALS

Recent reports show that FUS mutations cause impairment of proper DNA damage response,^13^, ^15^, ^25^ which was most strikingly seen in P525L, truncating mutations, and R521C. Therefore, we hypothesized that this might be reflected by increased abundancy of malignancies in FUS mutation carriers. Data for neoplasms were available for 40 patients, most of whom belonging to cohort 1. Among all included individuals, we identified only one patient who suffered from a malignancy prior to ALS, namely an acute lymphoblastic leukemia (ALL), which was diagnosed during his childhood (Table 1). This results in a prevalence for malignancies of 2.5% of the FUS‐ALS patients, which is not significantly different from the prevalence data from control population (1.6%) provided by data from the German cancer statistics from 2004^22^ (Fisher's exact test; *P* = 0.477). Considering that the study by Haberland et al. only covered a 5‐year prevalence, whereas our data can be understood as a lifetime prevalence, we recalculated with data from a register‐based study.^20^ Fang and colleagues did not detect a difference in malignancy burden between ALS cases and a healthy control population, but the rate for tumor prevalence in ALS cases was higher (10%) than in our study. Nevertheless, their ALS cohort and control patients did not differ significantly from our FUS‐ALS cohort regarding cancer prevalence (Fisher's exact test; *P* = 0.18).

Interestingly, the affected patient, who had a severe FUS frameshift mutation R495Qfs*527 leading to truncation of the NLS, received prophylactic whole‐brain radiation during his ALL therapy. Later on, he was diagnosed with a meningioma and eventually with ALS.

Conclusively, our data did not indicate an increased risk for malignancies in FUS‐ALS patients.

## Discussion

In this study, we provide the largest so far reported cohort of 186 FUS‐ALS patients and thereby enable a careful prediction of the clinical course in newly diagnosed patients and their families carrying the more common amino acid changes. FUS‐ALS patients showed a younger disease onset (median 39 years) than sporadic ALS patients.^10^, ^24^ This was, however, driven mainly by P525L and truncation/frameshift mutations leading to a higher frequency of bulbar onset and shorter survival. The less frequent Y526C mutation was also associated with particularly young onset, rapid disease progression, and atypical clinical signs as similarly demonstrated in a recent case report.^26^ Therefore, FUS mutations should be considered in early onset and atypical forms of MN impairment including sporadic patients^5^ with negative family history. We more often found de novo mutations in patients with a more drastic disease course and younger onset suggesting a possibly higher penetrance or lower chance to inherit the genetic change.

Our work extends previous work on genotype–phenotype correlations^7^, ^10^ by combining data from our patients with those FUS cases from the recent literature. This enabled us to identify patterns in the so far reported clinical heterogeneity by stratifying for certain mutations affecting the C‐terminus, which once more became clear to be the major cause of FUS‐ALS. Nevertheless, our study still lacks significant numbers of non‐NLS mutations to describe their phenotypes properly. Furthermore, other modifying factors than the mutation itself must play a role considering the sometimes very different disease parameters within families carrying the same mutations (Table 1).

There is evidence for a lower risk of cancer in several neurodegenerative disorders like Alzheimer's, Parkinson's, and Huntington's disease.^18^, ^19^, ^27^ On the other hand, patients with the ataxia‐telangiectasia (AT) syndrome present with both neurodegeneration and higher risk for lymphoreticular malignancies and breast cancer. This is due to homozygous loss‐of‐function mutations in the *ATM* gene leading to insufficient DNA double‐strand break repair,^28^, ^29^ which was also reported for *FUS* mutations.^15^

Thus, we addressed the clinical relevant question if FUS‐ALS patients have a higher prevalence of malignant neoplasms. The retrospective assessment in our cohort revealed a lifetime prevalence of 2.5%. This was not significantly higher^22^ or lower^20^ than in the general population or in sporadic ALS. Even though the sample size is small and the study retrospective with the chance of underestimating neoplasm incidence, we consider the available data being sufficient to rule out an obvious co‐occurrence of cancer as it is reported for AT syndromes.^28^ It is important to stress that the calculation was done using prevalence data not considering the lifetime morbidity risk for a cancer disease, which is strongly age‐dependent.^22^ The retrospective database analysis of *Fang et al.* might be more suitable when comparing to FUS‐ALS patients. FUS‐ALS is otherwise regarded to be the most frequent ALS subtype in juvenile patients (<35 years)^5^ suggesting that a lower number of cancer cases could be due to young age of affected individuals with aggressive disease course leading to death before the classically increased risk for malignancies with higher age. Therefore, it would be important to follow‐up the cases of unaffected family members of patients with evident *FUS* mutations and to compare with younger control cohorts.

Finally, there were only a few *FUS* mutations reported to influence DNA repair,^13^, ^14^, ^15^, ^25^ thus it is possible that the majority of patients not carrying those were not at risk for cancer which could not be addressed in our sample size. However, the most frequent FUS‐ALS mutation R521C was described to impair proper DNA damage response resulting in increased DNA double‐strand breaks evidently found in postmortem human tissue,^15^ but none of our 12 R521C patients with partially late disease onset showed evidence for neoplasms.

To our knowledge, our survey has assessed the largest FUS‐ALS patient collective so far. However, the rarity of the disease and the retrospective style has obvious limitations and warrants prospective validation. Still, we believe that our survey provides a sufficient cohort, which should be helpful when counseling patients and their families.

## Author Contributions

M.N. and A.H. concepted and designed the study including statistics and data acquisition of previously published and newly reported patients and manuscript drafting. K.P. and R.G. contributed patient data and helped designing the manuscript. V.D., A.J.K., E.A., P.D., F.W., P.C., P.A., J.W., J.P., S.C., A.H., A.C.L., F.P., and A.G. contributed patient data, critically reviewed the manuscript, and helped designing it.

## Conflict of Interest

The authors report no conflict of interest.

## Supporting information


**Figure S1** **.** Depiction of the individual age of onset of all patients (cohort 1 and 2) relative to the median (39), which illustrates the clinical heterogeneity of the disease.
**Table S1** **.** FUS‐ALS patients identified by pubmed review and novel patients
**Table S2** **.** Descriptive data of newly identified patients (cohort 1).
**Table S3** **.** Descriptive data of previously published cases (cohort 2).
**Table S4** **.** Descriptive data of the combined cohorts (cohort 1 + 2).
**Data S1** **.** Additional file providing raw data on published FUS‐ALS cases including their source and descriptive data. Figure S1 similarly to Figure 1 depicts the individual AoO but for all patients of the study.
