# Supplementary material for: Phenotypes and malignancy risk of different FUS mutations in genetic amyotrophic lateral sclerosis
Source: Ann Clin Transl Neurol. 2019 Nov 4;6(12):2384–94. doi: 10.1002/acn3.50930 (PMC6917314; doi:10.1002/acn3.50930)

**Suppl. Table 1. List of all patients including the respective source literature**

| Patient | Mutation/ aminoacid change | Age of Onset (AoO) | Duration (months to death) | Duration (onset to severe event) - OTSE | Site of onset | Source |
| --- | --- | --- | --- | --- | --- | --- |
| 1 | G156E | 33 | 11 | 11 | Bulbar | Ticozzi et al. 2009 |
| 2 | R234L | 66 | NA | NA | Spinal | Ticozzi et al. 2009 |
| 3 | R521C | 32 | 11 | NA | Bulbar | Ticozzi et al. 2009 |
| 4 | R521C | 25 | 60 | NA | Spinal | Ticozzi et al. 2009 |
| 5 | R521G | NA | NA | NA | Spinal | Ticozzi et al. 2009 |
| 6 | R514S | 36 | 22 | 22 | Spinal | Chio et al. 2009 |
| 7 | P525L | 21 | 12 | 12 | Bulbar | Chio et al. 2009 |
| 8 | R524S | 34 | 39 | 39 | NA | Kwiatkowski et al. 2009 |
| 9 | P525L | 22 | 6 | 6 | NA | Kwiatkowski et al. 2009 |
| 10 | S57del | 80 | 10 | 10 | Spinal | Belzil et al. 2009 |
| 11 | R521C | 26 | 27 | 27 | Spinal | Belzil et al. 2009 |
| 12 | R521H | 54 | 30 | 30 | Spinal | Belzil et al. 2009 |
| 13 | R521H | 32 | 72 | 72 | Spinal | Belzil et al. 2009 |
| 14 | G191S | 43 | 108 | 108 | Lower limbs | Corrado et al. 2010 |
| 15 | R216C | 50 | na | na | NA | Corrado et al. 2010 |
| 16 | G225V | 26 | 60 | 60 | Lower limbs | Corrado et al. 2010 |
| 17 | G230C | 65 | na | na | Lower limbs | Corrado et al. 2010 |
| 18 | R234C | 40 | 120 | 120 | Upper/lower limbs | Corrado et al. 2010 |
| 19 | G507D | 70 | na | na | NA | Corrado et al. 2010 |
| 20 | G507D | 50 | na | na | NA | Corrado et al. 2010 |
| 21 | R521C | 53 | 24 | 24 | Upper limbs | Corrado et al. 2010 |
| 22 | R521C | 34 | 7 | 7 | Upper limbs | Corrado et al. 2010 |
| 23 | R521H | 49 | 24 | 24 | Limbs | Van Damme et al. 2010 |
| 24 | R521H | 65 | 60 | 60 | Limbs | Van Damme et al. 2010 |
| 25 | R521H | 60 | 36 | 36 | Left arm | Van Damme et al. 2010 |
| 26 | R521H | 33 | NA | NA | Left leg | Van Damme et al. 2010 |
| 27 | Y66Y | 69 | NA | NA | Limb | Lai et al. 2010 |
| 28 | G507D | 41 | NA | NA | Limb | Lai et al. 2010 |
| 29 | R518G | 46 | NA | NA | Limb | Lai et al. 2010 |
| 30 | R521C | 34 | NA | NA | Limb | Lai et al. 2010 |
| 31 | R521H | 37 | NA | NA | Limb | Lai et al. 2010 |
| 32 | P525L | 55 | NA | NA | Bulbar | Lai et al. 2010 |
| 33 | R521C | 36 | 17 | 17 | Limb | Groen et al. 2010 |
| 34 | R521C | 29 | 34 | 34 | Limb | Groen et al. 2010 |
| 35 | R521H | 39 | 12 | 12 | Limb | Groen et al. 2010 |
| 36 | S462F | 63 | 100 | 100 | Limb | Groen et al. 2010 |
| 37 | G466Vfs*14 | 20 | 22 | 22 | Bulbar | DeJesus-Hernandez et al. 2010 |
| 38 | p.S402_P411delinsGGGG | 65 | 13 | 13 | Bulbar | DeJesus-Hernandez et al. 2010 |
| 39 | R524W | 55 | 36 | 36 | NA | Hewitt et al. 2010 |
| 40 | R524W | 60 | 38 | 38 | NA | Hewitt et al. 2010 |
| 41 | G174del | 62 | 26 | 26 | Bulbar | Hewitt et al. 2010 |
| 42 | G507D | 69 | 42 | 42 | NA | Hewitt et al. 2010 |
| 43 | G228dupG | 41 | 25 | 25 | Bulbar | Hewitt et al. 2010 |
| 44 | H517D | 42 | NA | NA | Bulbar (dysarthria) | Tsai et al. 2010 |
| 45 | R521H | 55 | NA | NA | Spinal (right hand) | Tsai et al. 2010 |
| 46 | R521H | 62 | 42 | 42 | Spinal, legs | Rademakers et al. 2010 |
| 47 | R521H | 39 | 42 | 42 | Spinal, legs | Rademakers et al. 2010 |
| 48 | G187S | 79 | 14 | 14 | Bulbar | Rademakers et al. 2010 |
| 49 | R495* | 31 | 18 | 18 | Bulbar | Waibel et al. 2010 |
| 50 | R510K | 39 | 96 | 96 | Spinal | Waibel et al. 2010 |
| 51 | R510K | 55 | 72 | 72 | Spinal | Waibel et al. 2010 |
| 52 | R495* | 28 | 24 | 24 | Bulbar | Calvo et al. 2014 |
| 53 | G497Afs*527 | 29 | 12 | 12 | Spinal | Yan et al. 2010 |
| 54 | K510Wfs*517 | 23 | 13 | 13 | Bulbar | Yan et al. 2010 |
| 55 | R495Efs*527 | 23 | 48 | 48 | Bulbar | Yan et al. 2010 |
| 56 | R495* | 24 | 12 | 12 | NA | Yan et al. 2010 |
| 57 | R521L | 71 | 12 | 12 | Spinal | Yan et al. 2010 |
| 58 | R524S | 48 | 10 | 10 | Spinal | Yan et al. 2010 |
| 59 | S96del | NA | 213 | 213 | NA | Yan et al. 2010 |
| 60 | P525L | 22 | 10 | 10 | Spinal | Bäumer et al. 2010 |
| 61 | P525L | 18 | 11 | 11 | Left upper arm | Bäumer et al. 2010 |
| 62 | c.1554_1557delACAG | 18 | 6 | 6 | Upper limbs | Bäumer et al. 2010 |
| 63 | R521C | 53 | 60 | 60 | Upper limb | Sproviero et al. 2012 |
| 64 | R521G | 32 | 27 | 27 | Upper limb | Sproviero et al. 2012 |
| 65 | P525L | 26 | 13 | 13 | Lower limb | Sproviero et al. 2012 |
| 66 | P525L | 45 | 42 | 42 | Upper limb | Sproviero et al. 2012 |
| 67 | R495* | 29 | NA | NA | Bulbar | Kwon et al. 2012 |
| 68 | G503Wfs*12 | 34 | NA | NA | Limb | Kwon et al. 2012 |
| 69 | X527YextX | 44 | NA | NA | Limb | Kwon et al. 2012 |
| 70 | c.1169-5A > G | 32 | NA | NA | Bulbar | Kwon et al. 2012 |
| 71 | G399V | 73 | NA | NA | Bulbar | Kwon et al. 2012 |
| 72 | p.228_230delGGG | 44 | NA | NA | Bulbar | Kwon et al. 2012 |
| 73 | p.230delG | 59 | NA | NA | Limb | Kwon et al. 2012 |
| 72 | p.228_230delGGG | 44 | NA | NA | Bulbar | Kwon et al. 2012 |
| 73 | p.230delG | 59 | NA | NA | Limb | Kwon et al. 2012 |
| 74 | G472Vfs*57 | 26 | 12 | 12 | NA | Hara et al. 2012 |
| 75 | G478LfsX23 | 21 | 23 | 23 | Bulbar | Waibel et al. 2013 |
| 76 | R495X | 31 | 18 | 18 | Bulbar | Waibel et al. 2013 |
| 77 | K510R | 55 | 72 | 72 | Spinal | Waibel et al. 2013 |
| 78 | K510R | 39 | 72 | 72 | Spinal | Waibel et al. 2013 |
| 79 | K510R | 41 | 84 | 84 | Spinal | Waibel et al. 2013 |
| 80 | K510R | 34 | > 240 alive | > 240 alive | Spinal | Waibel et al. 2013 |
| 81 | R514G | 41 | alive (> 5 years) | alive (> 5 years) | Bulbar | Waibel et al. 2013 |
| 82 | R521H | 46 | 36 | 36 | Spinal | Waibel et al. 2013 |
| 83 | G515Vfs*14 | 22 | 84 | 84 | Upper limb | Liu et al. 2017 |
| 84 | Q519Ifs*9 | 11 | 76 | 76 | Lower limb | Liu et al. 2017 |
| 85 | G486Pfs*30 | 15 | 73 (alive) | 73 (alive) | Lower limb | Liu et al. 2017 |
| 86 | R498Afs*32 | 20 | 12 | 12 | Lower limb | Liu et al. 2017 |
| 87 | K510M | 39 | 102 | 20 | NA | Mochizuki et al. 201 |
| 88 | G504WfsX515 | 24 | NA | NA | NA | Hariyanagi et al. 2016 |
| 89 | P525L | 21 | NA | NA | Bulbar | LeBlond et al. 2016 |
| 90 | P525L | 18 | 9 alive | not applicable | Bulbar | Hübers et al. 2015 |
| 91 | P525L | 20 | 15 alive | not applicable | Bulbar | Hübers et al. 2015 |
| 92 | P525L | 24 | 7 | 7 | Bulbar | Hübers et al. 2015 |
| 93 | R495Qfs*34 | 27 | 14 | 14 | Spinal | Hübers et al. 2015 |
| 94 | R521L | 30 | 40 | 40 | Spinal | Hübers et al. 2015 |
| 95 | R521H | 41 | 36 | 36 | Right arm | Hou et al. 2016 |
| 96 | P525dupY | 43 | 28 | 28 | Right arm | Hou et al. 2016 |
| 97 | R495X | 27 | 48 | 12 | Bulbar | Kim et al. 2015 |
| 98 | G504Wfs*12 | 31 | 48 | 48 | Limb | Kim et al. 2015 |
| 99 | R521H | 76 | 72 | 72 | Spinal | Blair et al. 2010 |
| 100 | R521H | 56 | 30 | 30 | Spinal | Blair et al. 2010 |
| 101 | R521H | 60 | alive (> 2 years) | not applicable | Spinal | Blair et al. 2010 |
| 102 | R521H | 49 | 24 | 24 | Spinal | Blair et al. 2010 |
| 103 | R521H | 56 | 60 | 60 | Spinal | Blair et al. 2010 |
| 104 | R521H | 52 | 48 | 48 | Spinal | Blair et al. 2010 |
| 105 | R521C | 47 | NA | NA | NA | Blair et al. 2010 |
| 106 | R521C | 48 | 12 | 12 | Spinal | Blair et al. 2010 |
| 107 | R521C | 27 | 24 | 24 | Spinal/bulbar | Blair et al. 2010 |
| 108 | R521C | 30 | NA | NA | Spinal | Blair et al. 2010 |
| 109 | R521C | 41 | alive (>5 years) | not applicable | Spinal | Blair et al. 2010 |
| 110 | R521C | 31 | 18 | 18 | NA | Blair et al. 2010 |
| 111 | R521C | 36 | alive (>1 year) | not applicable | NA | Blair et al. 2010 |
| 112 | R524W | 61 | 27 | 27 | NA | Hewitt et al. 2010 |
| 113 | R521C | 30 | 120 | 12 | spinal | Suzuki et al. 2010 |
| 114 | R521C | 38 | 108 | 26 | NA | Yamamoto-Watanabe et al. 2010 |
| 115 | G492Efs*527 | 17 | NA | NA | NA | Yamashita et al. 2012 |
| 116 | R514S | 24 | NA | NA | NA | Yamashita et al. 2012 |
| 117 | R495Qfs*527 | 19 | NA | NA | NA | Belzil et al. 2012 |
| 118 | P525L | 11 | not applicable | 14 | spinal | Conte et al. 2012 |
| 119 | R514S | 42 | 13 | 13 | Bulbar | Ito et al. 2011 |
| 120 | R521C | 30 | 120 | 12 | spinal | Ito et al. 2011 |
| 121 | P525L | 13 | 312 | 24 | spinal | Ito et al. 2011 |
| 122 | P525L | 13 | 20 | 17 | spinal | Huang et al. 2010 |
| 123 | M464I | 74 | 24 | 24 | NA | Nagayama et al. 2012 |
| 124 | G504Wfs*12 | 19 | NA | NA | spinal, right arm | Zou et al 2013 |
| 125 | P525L | 19 | NA | NA | spinal, right arm | Zou et al 2013 |
| 126 | R495* | 22 | not applicable | NA | Lower limbs | Zou et al 2013 |
| 127 | R521G | 49 | NA | NA | Upper limbs | Zou et al 2013 |
| 128 | R521L | 42 | 48 | 48 | Upper limb | Zou et al. 2012 |
| 129 | R521H | 45 | NA | NA | Upper limb | Zou et al. 2012 |
| 130 | R521H | 49 | 49 | 49 | Upper limb | Zou et al. 2012 |
| 131 | R521C | 43 | 72 | 72 | Spinal | Drepper et al. 2011 |
| 132 | R521C | 29 | 132 | 48 | Spinal | Drepper et al. 2011 |
| 133 | R522R | 66 | NA | NA | NA | Drepper et al. 2011 |
| 134 | R522R | 48 | NA | NA | NA | Drepper et al. 2011 |
| 135 | R522R | 52 | Na | Na | NA | Drepper et al. 2011 |
| 136 | G225V | 26 | NA | NA | bulbar | Bertolin et al. 2014 |
| 137 | G496Gfs*31 | 39 | NA | NA | spinal | Bertolin et al. 2014 |
| 138 | R521C | 51 | NA | NA | spinal | Bertolin et al. 2014 |
| 139 | G245V | 71 | 49 | 49 | spinal | Tarlarini et al. 2015 |
| 140 | P525L | 33 | 14 | 6 | Bulbar | Tarlarini et al. 2015 |
| 141 | R521C | 44 | 93 | 72 | spinal | Tarlarini et al. 2015 |
| 142 | R522G | 36 | 30 | 24 | spinal (respiratory) | Tarlarini et al. 2015 |
| 143 | G509D | 51 | 43 | 43 | spinal | Tarlarini et al. 2015 |
| 144 | R521C | 47 | 5 | 5 | spinal | Tarlarini et al. 2015 |
| 145 | R521C | 49 | 16 | 16 | spinal | Tarlarini et al. 2015 |
| 146 | P525L | 13 | 11 | 11 | NA | Kenna et al. 2013 |
| 147 | P525L | 21 | 17 | 17 | spinal | Kenna et al. 2013 |
| 148 | R524M | 32 | NA | NA | NA | Özoğuz et al. 2015 |
| 149 | R524M | 53 | NA | NA | NA | Özoğuz et al. 2015 |
| 150 | P525L | 14 | NA | NA | NA | Özoğuz et al. 2015 |
| 151 | R521L | 58 | 7 | 7 | arms | This study |
| 152 | R521C | 61 | 60 | 60 | arms | This study |
| 153 | P525L | 22 | not applicable | 7 | arms | This study |
| 154 | R495Qfs*527 | 31 | 18 | 18 | bulbar | This study |
| 155 | R521C | 62 | 48 | 48 | legs left | This study |
| 156 | R521H | NA | NA | NA | NA | This study |
| 157 | R521H | 46 | 37 | 37 | arms | This study |
| 158 | R521H | 43 | not applicable | 61 | arms right | This study |
| 159 | G478Lfs*23 | 23 | 19 | 19 | Legs right | This study |
| 160 | R521H | NA | NA | NA | NA | This study |
| 161 | R521H | NA | NA | NA | NA | This study |
| 162 | R521H | NA | NA | NA | NA | This study |
| 163 | R495* | censored | 10 | 10 | NA | This study |
| 164 | Q23L | 35 | 175 | 175 | right hand | This study |
| 165 | M254I | 63 | NA | 72 | NA | This study |
| 166 | R521C | NA | NA | NA | NA | This study |
| 167 | R521C | NA | NA | NA | NA | This study |
| 168 | R521H | 44 | 38 | 38 | arms (left) | This study |
| 169 | R521H | 71 | 29 | 29 | arms (left) | This study |
| 170 | R521H | 38 | 31 | 31 | legs (right) | This study |
| 171 | P525L | 17 | 15 | 15 | legs (bilateral) | This study |
| 172 | R521C | 39 | 20 | 20 | right arm | This study |
| 173 | R521C | 54 | 27 | 27 | left arm | This study |
| 174 | R521C | 39 | 19 | 19 | right leg | This study |
| 175 | R521C | 38 | 25 | 25 | legs | This study |
| 176 | Y526C | 33 | 16 | 16 | dropped head | This study |
| 177 | R521C | 39 | 20 | 20 | bulbar | This study |
| 178 | R521C | NA | NA | NA | NA | This study |
| 179 | Y526C | 24 | not applicable | 9 | bulbar | This study |
| 180 | R521C | 40 | 13 | 13 | arms (left) | This study |
| 181 | R521C | 27 | 13 | 13 | arms/ shoulders | This study |
| 182 | D502Tfs*27 | 19 | NA | 17 | arms (right) | Hübers et al. 2015 |
| 183 | P525L | 17 | 27 | 27 | bulbar | This study |
| 184 | K510R | 59 | not applicable | not applicable | legs (left) | This study |
| 185 | G509D | 41 | not applicable | not applicable | spinal (legs) | This study |
| 186 | R521H | 40 | not applicable | not applicable | legs (right) | This study |

**Suppl. Table 2. Descriptive data on cohort 1**

|  | N | Minimum | Maximum | mean | median | SEM | StDev |
| --- | --- | --- | --- | --- | --- | --- | --- |
| Age of onset (AoO, years) | 28 | 13 | 71 | 39.9 | 39 | 2.9 | 15.3 |
| Onset to severe event (OTSE, months) | 26 | 7 | 175 | 32.5 | 22.5 | 6.6 | 33.7 |

**Suppl. Table 3. Descriptive data on cohort 2**

|  | N | Minimum | Maximum | mean | median | SEM | StDev |
| --- | --- | --- | --- | --- | --- | --- | --- |
| Age of onset (AoO, years) | 148 | 11 | 80 | 39.7 | 39 | 1.4 | 16.5 |
| Onset to severe event (OTSE, months) | 98 | 5 | 213 | 35.3 | 24 | 3.2 | 31.2 |

**Suppl. Table 4. Descriptive data on cohort 1+2**

|  | N | Minimum | Maximum | mean | median | SEM | StDev |
| --- | --- | --- | --- | --- | --- | --- | --- |
| Age of onset (AoO, years) | 176 | 11 | 80 | 39.7 | 39 | 1.2 | 16.3 |
| Onset to severe event (OTSE, months) | 124 | 5 | 213 | 34.7 | 24 | 2.8 | 31.7 |

**Supplemental Figure 1**


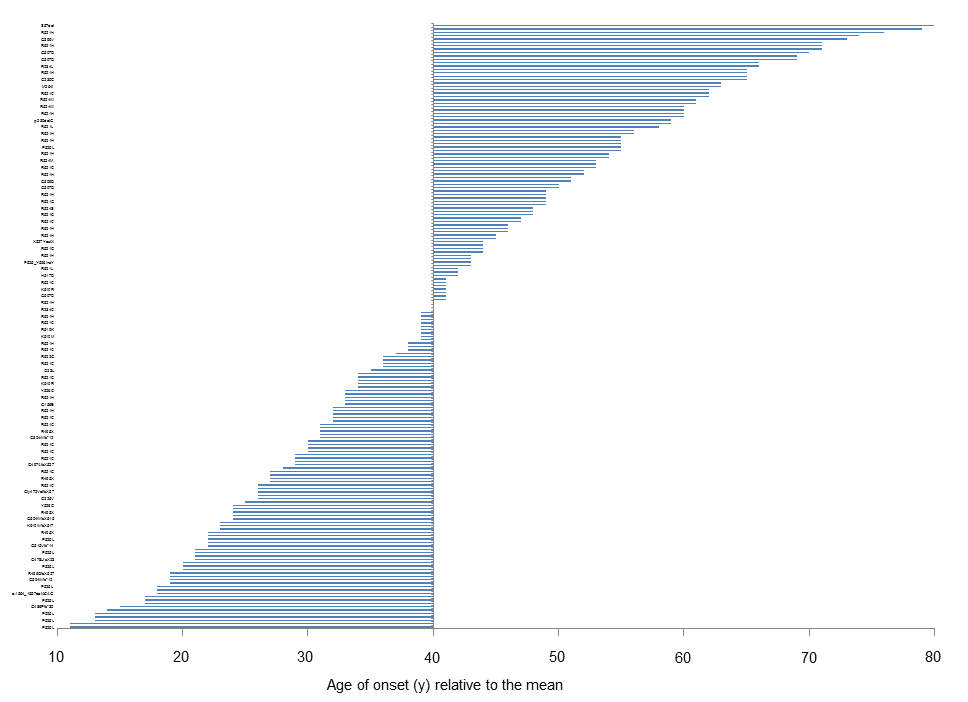

Supplement: Supplementary file 1 — Figure S1 . Depiction of the individual age of onset of all patients (cohort 1 and 2) relative to the median (39), which illustrates the clinical heterogeneity of the disease. Table S1 . FUS‐ALS patients identified by pubmed review and novel patients Table S2 . Descriptive data of newly identified patients (cohort 1). Table S3 . Descriptive data of previously published cases (cohort 2). Table S4 . Descriptive data of the combined cohorts (cohort 1 + 2). Data S1 . Additional file providing raw data on published FUS‐ALS cases including their source and descriptive data. Figure S1 similarly to Figure 1 depicts the individual AoO but for all patients of the study. [file ACN3-6-2384-s001.docx]
